# Molecular Modeling Studies on Carbazole Carboxamide Based BTK Inhibitors Using Docking and Structure-Based 3D-QSAR

**DOI:** 10.3390/ijms19041244

**Published:** 2018-04-19

**Authors:** Rui Li, Yongli Du, Zhipei Gao, Jingkang Shen

**Affiliations:** 1 School of Chemistry and Pharmaceutical Engineering, Qilu University of Technology (Shandong Academy of Sciences), 3501 Daxue Road, Jinan 250353, China; 18364173762@163.com (R.L.); gaomills@163.com (Z.G.); 2 State Key Laboratory of Drug Research, Shanghai Institute of Materia Medica, Chinese Academy of Sciences, 555 Zu Chong Zhi Road, Shanghai 201203, China; jkshen@simm.ac.cn

**Keywords:** rheumatoid arthritis (RA), Broton’s tyrosine kinase (BTK), carbazole carboxamide derivatives, 3D-QSAR, comparative molecular field analysis (CoMFA), comparative molecular similarity indices analysis (CoMSIA)

## Abstract

Rheumatoid arthritis (RA) is the second common rheumatic immune disease with chronic, invasive inflammatory characteristics. Non-steroidal anti-inflammatory drugs (NSAIDs), slow-acting anti-rheumatic drugs (SAARDs), or glucocorticoid drugs can improve RA patients’ symptoms, but fail to cure. Broton’s tyrosine kinase (BTK) inhibitors have been proven to be an efficacious target against autoimmune indications and B-cell malignancies. Among the current 11 clinical drugs, only BMS-986142, classified as a carbazole derivative, is used for treating RA. To design novel and highly potent carbazole inhibitors, molecular docking and three dimensional quantitative structure–activity relationship (3D-QSAR) were applied to explore a dataset of 132 new carbazole carboxamide derivatives. The established comparative molecular field analysis (CoMFA) (*q*^2^ = 0.761, *r*^2^ = 0.933) and comparative molecular similarity indices analysis (CoMSIA) (*q*^2^ = 0.891, *r*^2^ = 0.988) models obtained high predictive and satisfactory values. CoMFA/CoMSIA contour maps demonstrated that bulky substitutions and hydrogen-bond donors were preferred at R_1_ and 1-position, respectively, and introducing hydrophilic substitutions at R_1_ and R_4_ was important for improving BTK inhibitory activities. These results will contribute to the design of novel and highly potent BTK inhibitors.

## 1. Introduction

Rheumatoid arthritis (RA) is an autoimmune destructive disease by affecting the joints, causing progressive, symmetric, erosive destruction of cartilage and bone [1]. RA has affected about 24.5 million people as of 2015, and the condition newly develops in approximately 1% of the population each year [2]. Two main classes of traditional medications were used for treatment of RA: first-line drugs (involved non-steroidal anti-inflammatory drugs (NSAIDs) and corticosteroids) and second-line drugs (also referred to as disease-modifying anti rheumatic drugs or disease-modifying anti rheumatic drugs (DMARDs)) [3]. However, these two classes of medicine possess some serious side effects, such as increased susceptibility to bruising, abdominal pain, and even risk of infections and bleeding [4]. Therefore, it is increasingly crucial to develop novel drugs with improved efficacy and safety in RA treatment.

**Figure 1:**
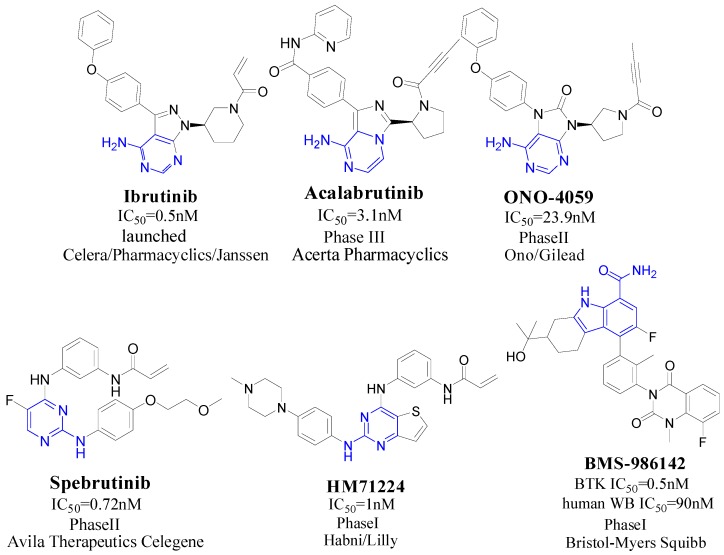
The chemical structures of several Broton’s tyrosine kinase (BTK) inhibitors that have entered into clinical trials.

Broton’s tyrosine kinase (BTK) is a member of the Tyrosine-protein kinase (TEC) kinase family and plays a critical role in the B-cell development and activation through mediating the downstream signaling cascade of B-cell receptors (BCRs) [5,6]. The increase in BTK expression can cause the chronic activation of the BCR signaling pathway, which affects B-cell proliferation and differentiation [7]. As a result, it can cause a lack of antibodies in the body, which finally gives rise to RA and other inflammatory diseases [8]. Therefore, inhibiting BTK activities to keep the normal function of the BCR signaling pathway is an effective way to treat RA. Recently, BTK inhibitors have been of increased interest in the clinical study of B-cell tumors and immune disease. Ibrutinib [8,9], acalabrutinib [10], ONO-4059 [11], spebtutinib [12], HM71224 [13], and BMS-986142 [14] have advanced into clinical trials, and their reported chemical structures are shown in Figure 1. As candidate drugs for treating RA, only BMS-986142 has advanced into Clinical Phase I with improved oral exposure, kinase selectivity, and high BTK potency [15]. Compared with NSAIDs and DMARDs, BMS-986142 has advantages of increased safety and efficacy as well as the less dependence on medication [16]. Therefore, exploring novel and highly potent BTK inhibitors for RA treatment is an important and promising prospect.

Here we report on molecular modeling studies performed by comparative molecular field analysis (CoMFA) [17] and comparative molecular similarity indices analysis (CoMSIA) [18] modules, as well as docking results, to investigate the three-dimensional quantitative structure–activity relationship (3D-QSAR) between carbazole inhibitors (BMS-986142 analogues) and BTK.

## 2. Results and Discussion

### 2.1. Molecular Docking

**Figure 2:**
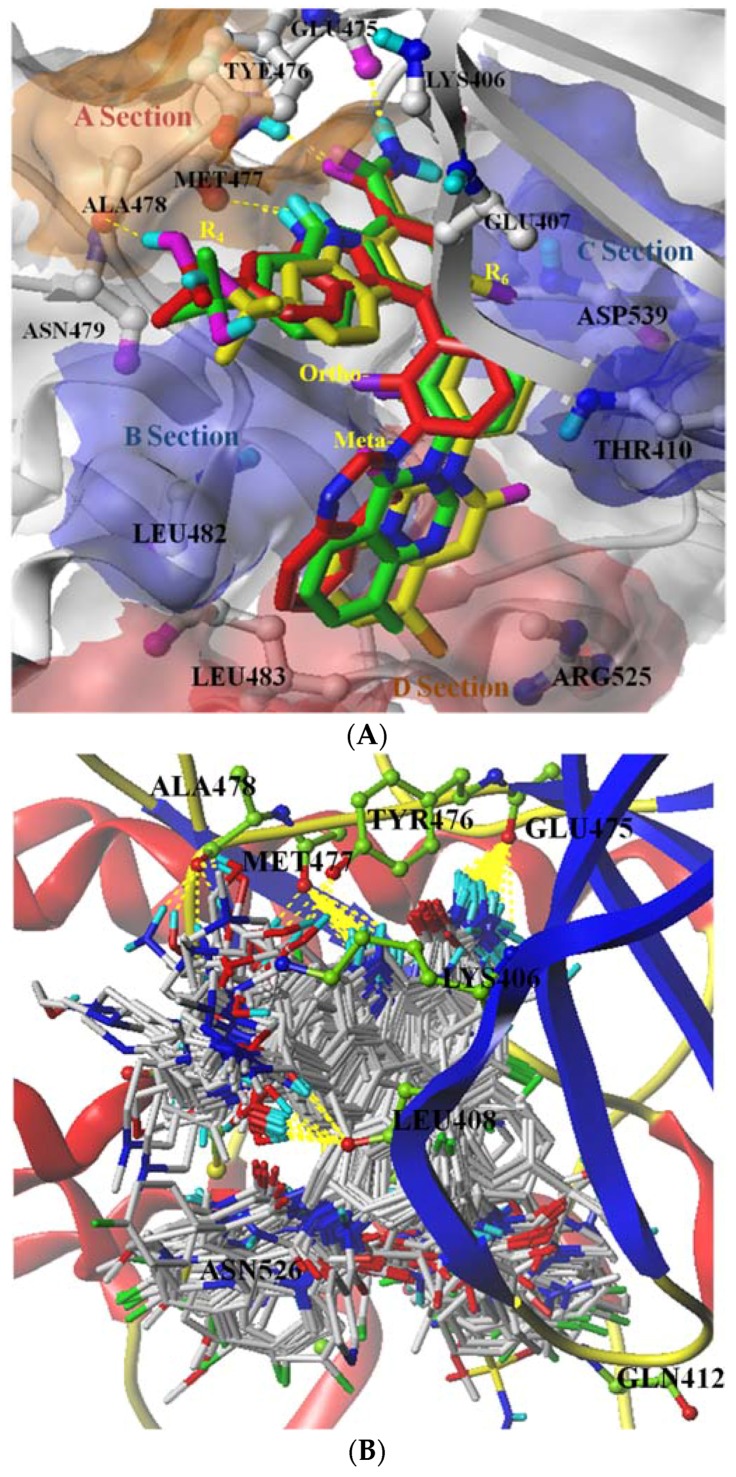
(**A**) The binding pose prediction of **76** (green) compared to ligand (red) found in an X-ray crystal structure; the position of **79** (yellow) in the active site of the protein and the binding pocket of BTK enzyme. (**B**) Docking-based alignment of dataset molecules. Hydrogen bonds are represented as yellow dotted lines, and main protein residues are labeled with ball and stick forms. Section A: hinge region; Sections B and C: hydrophobic pocket; Section D: floor loop.

The aim of the molecular docking was to predict the binding affinity and interactions of carbazoles known to modulate the activity of BTK. The accuracy of the docking program was confirmed by comparing the predicted compound (**76**, green) and ligand (red) extracted from the crystal structure of BTK (Protein Data Bank ID: 5JRS). The result, revealing excellent agreement, is shown in Figure 2A and confirms that the selected experimental parameters and procedures used for molecular docking and alignment were reasonable. As depicted in Figure 2A, the common carbazole rings of **76** and **79** as well as experimental ligand were in the same position and mainly interacted with residues Glu475, Tyr476, and Met477.

To explain the binding mode, **79** (IC_50_ = 0.22 nM) was selected for more detailed analysis, since it was the most representative inhibitor in the active site of the protein. Based on Figure 2A, the carbazole ring of **79** interacted with −C = O and N–H of Met 477 and −C = O of Gly475 by hydrogen bonds in the hinge region, and interacted with the benzene ring of Tyr 466 by a conjugate effect; among them, Gly475 and Met477 [19] are two significant gatekeeper residues in BTK enzyme. The hydroxyl group at R_4_ also had a hydrogen-bond interaction with Ala478. Chlorine atom at R_6_ formed a hydrophobic interaction with Glu407 and Asp539. The benzene ring’s ortho-groups at R_1_ also interacted with Cys527 and Leu528 through a hydrophobic effect. At the bottom of the pocket, a substituent at the meta-position of the benzene ring was well filled in a floor loop formed by Asn484, Leu483, and Arg525. All these action characteristics proved that **79** was the most active molecule in the dataset.

As shown in Figure 2B, the selected 132 molecules demonstrate similar features after they are aligned on the common substructure and interact with Gly475 and Met477 through hydrogen-bond actions. The activities factors are groups at R_4_ trending toward different directions and groups at R_6_ forming hydrophobic interactions with different residues. Substituents at R_1_ occupied in sites of the floor loop area are also different. These diverse elements resulted in the selected 132 molecules used to perform molecular modeling studies possessing multiple IC_50_ values.

### 2.2. 3D-QSAR Analysis Studies

The aligned dataset was subjected to establish 3D-QSAR modeling using partial least squares (PLS) statistics with different field contribution values. In order to select the best field combination model and avoid the over-fitting problem, the stability statistics including cross-validated correlation coefficient (*q*^2^), non-cross-validated correlation coefficient (*r*^2^), a standard error of estimate (SEE), an optimum number of components (NOC), and *F* statistical values were taken into consideration. As a rule of thumb, *q*^2^ and *r*^2^ should have higher values, while SEE should have smaller error values. Therefore, reasonable CoMFA (*q*^2^ = 0.761, NOC = 6, *r*^2^ = 0.933) and CoMSIA (*q*^2^ = 0.891, NOC = 9, *r*^2^ = 0.988) models were developed for the selected training set and the test set. The detailed statistical summary of the CoMFA and CoMSIA analysis are shown in Table 1.

A reasonable CoMFA model was established on the basis of satisfactory statistical values including q^2^, r^2^, and SEE values (0.761, 0.933, and 0.202, respectively). When steric, electrostatic, hydrophobic, and H-bond acceptor and donor fields were all employed in the CoMSIA model, q^2^, r^2^, and SEE values also acquired good results (0.891, 0.988, and 0.088, respectively), which confirmed that the CoMSIA model was reliable and reasonable.

### 2.3. Contour Map Analysis

Contour maps for CoMFA and CoMSIA were generated to visualize the information in 3D-QSAR models. The maps of the 3D-QSAR models based on PLS analysis provided a comprehensive understanding of the key structural requirements responsible for the biological activity and are depicted in the following.

#### 2.3.1. CoMFA Contour Map Analysis

**Figure 3:**
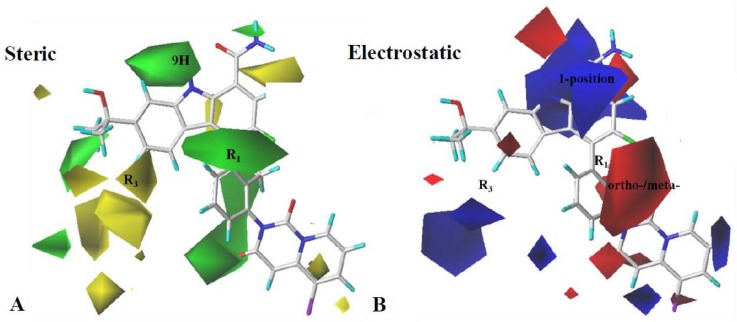
CoMFA StDev*Coeff contour maps. (**A**) Steric contour map (green: favored; yellow: disfavored). (**B**) Electrostatic contour map (blue: favored; red: disfavored). Compound **79** is shown as a capped sticks model.

CoMFA contour maps are vividly displayed in different color areas and illustrate whether the substituted groups are reasonable. Steric contour maps and electrostatic contour maps are shown in Figure 3A,B compared with **79**.

In the CoMFA steric contour map (Figure 3A), green represents favored bulky groups and yellow represents the opposite. Green contour maps appeared at 9H of carbazole and R_1_, indicating that more bulky groups in these regions could improve activity. This possibly explained that inhibitory activity of **53** (IC_50_ = 18 nM), **54** (IC_50_ = 18 nM), and **55** (IC_50_ = 17 nM) with a methyl at the benzene ring of R_1_ was twentyfold more potent compared with **127** (IC_50_ = 390 nM) with a hydrogen atom at this position. Besides, a yellow contour at R_3_ suggests that adding a bulky substitution in this region can decrease inhibitory activity, which may explain why the activities of **101**–**104** (IC_50_: 110–461 nM) with an added morpholinone or piperazinone group at R_3_ dropped sharply.

In the CoMFA electrostatic contour maps (Figure 3B), blue contours located near 1-position and R_3_ imply that positive substitutions in these region can increase the activity of the inhibitors. This may explain why **104** (IC_50_ = 110 nM) with a piperazin substituent at R_3_ was more potent than **102** (IC_50_ = 308 nM) with morpholin in the same position. Inversely, the red contour in the ortho- and meta-positions of the benzene ring at R_1_ suggested that negative atoms can increase the activity. This was in accordance with the fact that **84** (IC_50_ = 032 nM), **87** (IC_50_ = 0.25 nM), **129** (IC_50_ = 0.4 nM), and **130** (IC_50_ = 0.9 nM) possessing nitrogen (negative) atoms at R_1_ demonstrated high BTK inhibition activity.

#### 2.3.2. CoMSIA Contour Map Analysis

CoMSIA StDev*Coeff contour map analysis of steric, electrostatic, hydrophobic, and H-bond donor and H-bond acceptor fields are revealed in the following images, with **79** as the template molecule in the active site of BTK.

**Figure 4:**
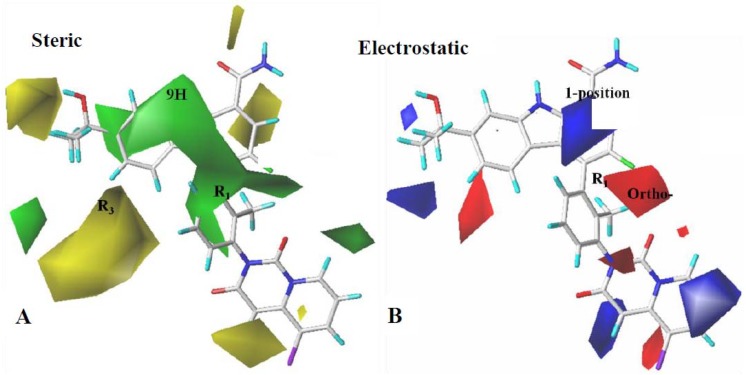
CoMSIA StDev*Coeff contour maps. (**A**) Steric contour map (green: favored; yellow: disfavored). (**B**) Electrostatic contour map (blue: favored; red: disfavored). Compound **79** is shown as a capped sticks model.

In the CoMSIA steric contour map (Figure 4A), the carbazole ring of **79**, sheathed by a giant green block, indicates that the bulky groups here can increase the activity. Yellow contours near the extensional area of R_3_ suggest the unfavorable influence of bulky groups. In Figure 4B, the electron-donating group and electron-withdrawing group covered by blue and red contours were represented at 1-position and ortho-position of the benzene ring at R_1_, respectively. Compared to the steric/electrostatic contour maps of CoMFA and CoMSIA, they are very similar, except that the largest green field also involved an outstretched space in the carbazole scaffold, which means that adding bulky groups to this region improved activity.

**Figure 5:**
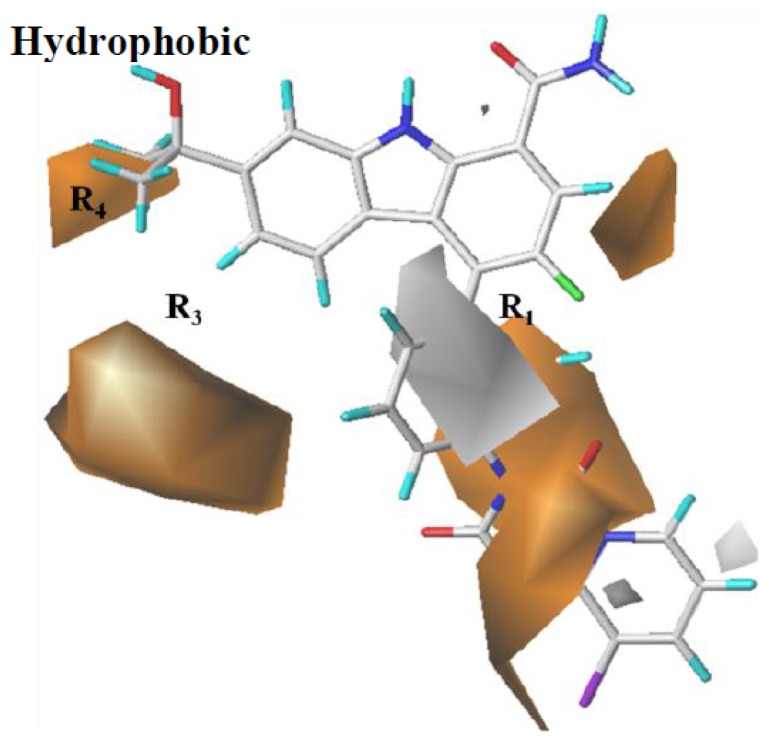
CoMSIA StDev*Coeff contour maps: Hydrophobic contour map (orange: favored; white: disfavored). Compound **79** is shown as a capped sticks model.

The hydrophobic contour map from CoMSIA is shown in Figure 5. Orange contours near the benzene ring of R_1_ and the hydrocarbyl of R_4_, as well as the extension space of R_3_, indicate that the hydrophobic groups in those areas are beneficial for inhibitory activities. This is consistent with the fact that **95**–**100** (IC_50_: 0.35–2.0 nM), possessing halogen and hydrocarbyl substituents in these areas, have more potent activities than **54** (IC_50_ = 18 nM) and **55** (IC_50_ = 17 nM) with the hydroxyl and amino groups. White contours around R_1_ reveal that the hydrophobic groups here do not help to enhance the activity. Hence, **121** (IC_50_ = 16 nM), **122** (IC_50_ = 15 nM), **124** (IC_50_ = 17 nM), and **125** (IC_50_ = 16 nM), possessing aromatic halogen substitutions at this position, held lower activity levels than **129**–**132** (IC_50_: 0.4–1.0 nM).

**Figure 6:**
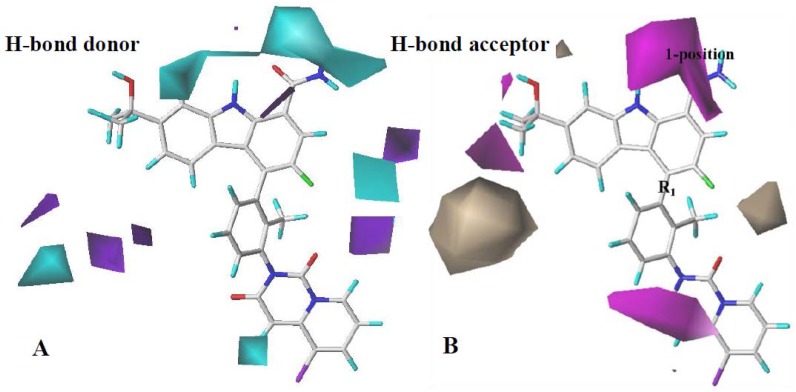
CoMSIA StDev*Coeff contour maps. (**A**) H-bond donor map (cyan: favored; purple: disfavored). (**B**) H-bond acceptor map (magenta: favored; brown: disfavored). Compound **79** is shown as a capped sticks model.

The H-bond donor and acceptor of the CoMSIA contour map are shown in Figure 6A,B, respectively. The remarkable cyan contour on the top of the carbazole ring implies that the presence of hydrogen-bond donor groups might enhance bioactivity. This could be validated if it is found that **1**–**132** possess hydrogen atoms as hydrogen-bond donor groups in the same positions. The magenta contours around 1-position and meta-position of the benzene ring at R_1_ show that H-bond acceptor groups in these places can increase the activity of inhibitors. This might explain why **74** (IC_50_ = 0.79 nM) and **75** (IC_50_ = 1.2 nM) with two carbonyl substituents at R_1_ displayed better IC_50_ values than **1** (IC_50_ = 44 nM).

### 2.4. Model Validation of CoMFA and CoMSIA Models

**Figure 7:**
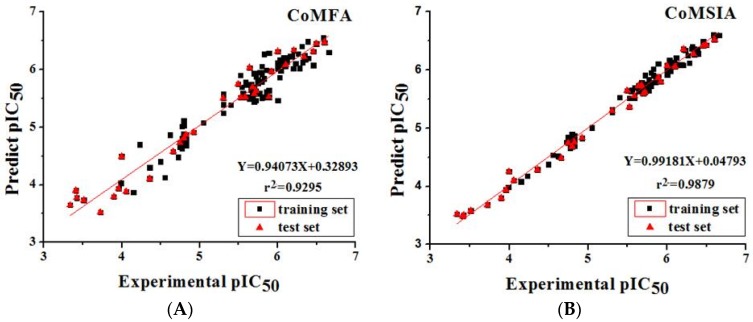
Correlation between the predicted and experimental activities of the training and test set compounds. (**A**) The scatter plot of CoMFA. (**B**) The scatter plot of CoMSIA. Black squares represent the training set; red triangles represent the test set.

The experimental and predicted activity values of CoMFA and CoMSIA models are depicted in Table 2, and their scatter plots are shown in Figure 7.

Based on the above data, the correlation coefficient between the predicted and experimental activities generated by the CoMFA models were 0.94073 and its analytical error was 0.32893, which confirmed that the established CoMFA models are reliable and reasonable. Similarly, the correlation coefficient and analytical error of the CoMSIA model were 0.99181 and 0.04793, respectively, and these two values verify that the CoMSIA models are accurate and reliable. Both CoMFA and CoMSIA models can be further used to predict activities of newly designed inhibitors.

## 3. Materials and Methods

### 3.1. Collection of the Dataset

A series of carbazole-carboxamide-based BTK inhibitors (BMS-986142 analogues) were used for the study. The 132 selected molecules [14,20,21,22,23] had a basic tricyclic skeleton and a similar binding mode with the BTK enzyme, which could be well superimposed in the alignment module. These BMS-986142 analogues were evenly distributed in an inhibitory activity range from 0.1 to 1000 nM. These compounds were optimized by energy minimization with a tripos force field in Sybyl-X 2.0 [24] and generated three-dimensional conformations after docking into the BTK-enzyme-binding site. The biological data expressed as IC_50_ values were converted into pIC_50_ (−log IC_50_) values, which were used as dependent variables in the following QSAR analyses [25]. The selected 132 BTK inhibitors were divided into a test set consisting of 32 molecules for model validation and a training set including 100 compounds for model generation. Thirty-two compounds in the test set were selected randomly and included compounds with a uniformly distributed range of pIC_50_ values from 3.336 to 6.658, covering more than 3 log units, which is fit for 3D QSAR studies [26]. The conformation of the most active compound, **79**, was selected as a template structure to sketch the rest of the molecules [27]. The complete dataset (1–132) taken for study is shown in Table 3.

### 3.2. Preparation of Protein

The crystal structure of BTK with high resolution was retrieved from the protein data bank (PDB ID: 5JRS) [20]. This crystal structure was prepared using a protein preparation module in Sybyl-X 2.0. Ligand and water molecules were removed. Furthermore, polar hydrogen atoms were added for investigating interactions between inhibitors and BTK.

### 3.3. Molecular Docking and Alignment

**Figure 8:**
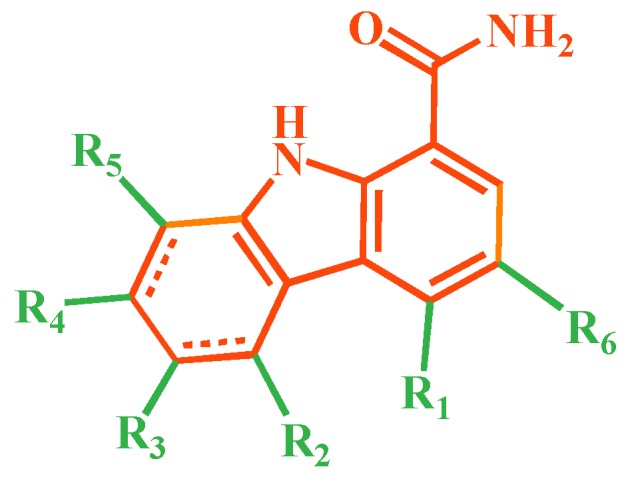
The common scaffold of the dataset.

The molecular dockings of **1**–**132** were performed using Surflex-Dock (SFXC) module with default parameters, except that the maximum number of per molecular conformation was defined as 40 to ensure that the docked conformations in the BTKBTK-binding site were reasonable. The rational docked conformations of the compound in the protein-binding site were picked up from the clustered docking poses according to the principle of low energy and rational conformation [28]. The most potent compound, **79**, with the rational conformation possessing the lowest energy, was chosen as the reference molecule. Rational conformations of the remaining inhibitors in the dataset based on the interactions with the BTK-enzyme-binding site were aligned on the common substructure of the reference compound (Figure 8). After the conformations were aligned in the BTKBTK-binding site, all selected conformations were conserved as a database file, which was used for 3D-QSAR study.

### 3.4. 3D-QSAR Analysis Studies

3D-QSAR analyses performed by the QSAR command bar of SYBYL X-2.0 (Tripos (DE), Inc., St. Louis, MO, USA) were carried out in the form of molecular spreadsheets to create CoMFA and CoMSIA fields from the database file acquired after molecular docking. The CoMFA [17] fields, including steric (S) and electrostatic (E) fields, were calculated under default settings with energy cutoff values of 30 kcal/mol. With the exception of the same fields in CoMFA, the CoMSIA [18] fields also containing hydrophobic (H) and hydrogen-bond donor (D) and acceptor (A) fields were derived using the same method as that of the CoMFA calculations. Both CoMFA and CoMSIA analyses were calculated in the standard settings with an attenuation factor α of 0.3. After 3D-QSAR analyses, the standard contour maps for both CoMFA and CoMSIA to visualize the results were developed using the field type StDev*Coeff.

### 3.5. Model Validation

All the developed CoMFA and CoMSIA models were checked for stability and robustness using the internal and external test set validations. Internal validation was carried out using a PLS [29] approach of cross-validation method to inspect the predictability of the dataset. The external test set containing 32 molecules not included in the model building was applied to verify the accuracy of the predictive abilities of the derived 3D-QSAR models. In the PLS approach, leave-one-out (LOO) method analysis generated the cross-validated q^2^ and the optimum number of components. The final CoMFA and CoMSIA models were developed using the obtained optimal number of components without cross-validation analysis. When the values of the coefficients fall between 1.0 and 0.5 [30], an accurate model is accepted. Furthermore, for better evaluation of the accuracy and robustness of the developed models, non-cross-validation analysis was employed to yield the conventional correlation coefficient *r*^2^ and the *F*-test value (*F*).

## 4. Conclusions

**Figure 9:**
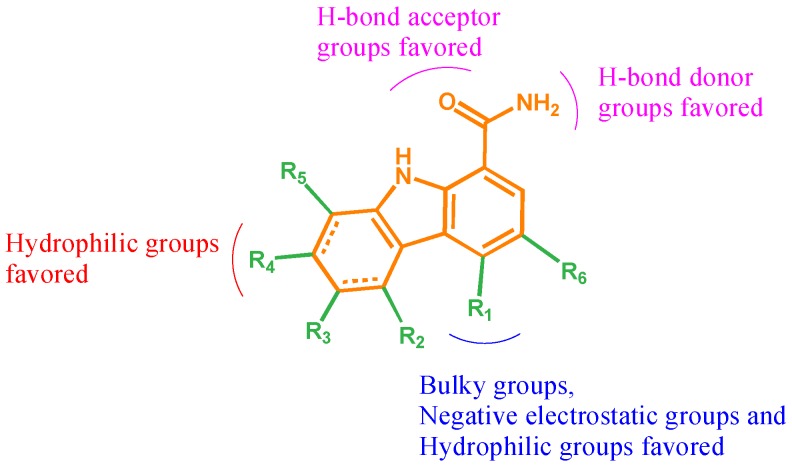
The structure–activity relationship (SAR) summarized based on our work.

A 3D-QSAR study on carbazole inhibitors based on a common scaffold was conducted with the generation of rational docking conformations and CoMFA/CoMSIA models. The reasonable CoMFA (*q*^2^ = 0.761, *r*^2^ = 0.933) and CoMSIA (*q*^2^ = 0.891, *r*^2^ = 0.988) models displayed satisfactory correlations and predictive abilities. CoMFA and CoMSIA contour maps provided information (shown in Figure 9) indicating that structural optimization for improving activities can be predominantly considered by adding bulky negative electrostatic groups and hydrophilic groups at R_1_, by increasing hydrophilic groups at R_4_, and by raising H-bond donor and acceptor substituents at 1-position. Moreover, the predicted ability of 3D-QSAR models was validated for application in predicting the activities of newly designed compounds and further provided a valuable clue in the design of novel carbazole inhibitors for RA treatment.

## Figures and Tables

**Table 1:** Detailed statistical summary of the comparative molecular field analysis (CoMFA) and comparative molecular similarity indices analysis (CoMSIA) models.

**CoMFA**	**NOC**	***q*^2^**	***r*^2^**	**SEE**	***F* Value**	**Field Contributions**				
						**S**	**E**	**H**	**D**	**A**
S+E	6	0.761	0.933	0.202	291.45	0.46	0.54	-	-	-
**CoMSIA**	**NOC**	***q*^2^**	***r*^2^**	**SEE**	***F* Value**	**Field Contributions**				
						**S**	**E**	**H**	**D**	**A**
S+E	5	0.851	0.941	0.188	404.01	0.198	0.802	-	-	-
S+E+H	7	0.862	0.972	0.132	606.51	0.110	0.554	0.336	-	-
S+E+D	4	0.863	0.930	0.205	420.26	0.117	0.515	-	0.367	-
S+E+A	7	0.863	0.974	0.127	657.51	0.122	0.535	-	-	0.342
S+E+H+D	9	0.875	0.985	0.095	920.97	0.069	0.424	0.235	0.272	-
S+E+H+A	9	0.880	0.986	0.095	923.65	0.073	0.411	0.254	-	0.262
S+E+D+A	10	0.878	0.985	0.092	1031.44	0.078	0.400	-	0.270	0.253
S+E+H+D+A	9	0.891	0.988	0.088	1076.36	0.053	0.342	0.193	0.208	0.203

*q*^2^: cross-validated correlation coefficient; NOC: optimum number of components; *r*^2^: non cross-validated correlation coefficient; SEE: standard error of estimation; *F* value: *F*-test value. S = steric; E = electrostatic; H = hydrophobic; A = acceptor; D = donor. Final chosen model for CoMSIA analysis is indicated in bold font.

**Table 2:** The experimental and predicted activity values for the developed models.

**Training Set Compounds**	**pIC_50_**	**CoMFA**		**CoMSIA**	
		**Predicted**	**Residuals**	**Predicted**	**Residuals**
**1**	4.3565	4.304	0.0525	4.286	0.0705
**3**	5.6778	5.554	0.1239	5.730	−0.0526
**4**	5.8239	5.526	0.2981	5.790	0.0344
**5**	5.6576	5.649	0.0082	5.673	−0.0157
**6**	5.7447	5.758	−0.0135	5.753	−0.008
**7**	5.6576	5.524	0.1337	5.697	−0.0393
**8**	5.6576	5.751	−0.0938	5.659	−0.0011
**9**	5.6576	5.735	−0.0776	5.656	0.0015
**10**	5.6383	5.493	0.1456	5.640	−0.0015
**11**	5.7959	6.058	−0.2625	5.957	−0.1615
**12**	6.0969	6.060	0.0366	6.102	−0.0048
**13**	5.6990	5.631	0.0675	5.653	0.0459
**14**	6.0000	6.042	−0.0422	5.925	0.0755
**15**	6.1551	6.234	−0.0789	6.087	0.0681
**17**	5.8861	5.641	0.2452	5.885	0.0008
**18**	5.6990	5.583	0.1156	5.704	−0.0049
**19**	5.7447	5.821	−0.0766	5.761	−0.0162
**20**	5.5376	5.698	−0.16	5.638	−0.1001
**22**	5.8539	5.615	0.2385	5.855	−0.0012
**23**	5.7213	5.465	0.2565	5.751	−0.0294
**24**	5.7959	5.917	−0.1211	5.731	0.0644
**25**	5.5229	5.901	−0.3785	5.516	0.0073
**26**	5.7213	5.876	−0.1547	5.908	−0.1865
**28**	6.0924	6.208	−0.1156	6.099	0.0066
**29**	6.2076	6.337	−0.1291	6.36	−0.1519
**31**	5.5528	5.597	−0.0445	5.654	−0.1009
**32**	5.0458	5.081	−0.0354	5.010	0.0357
**33**	5.5850	5.744	−0.1586	5.518	0.0669
**34**	5.8239	6.272	−0.4485	6.017	−0.1929
**36**	5.7695	5.951	−0.1814	5.642	0.1275
**37**	6.2007	6.017	0.1837	6.080	0.1203
**38**	6.2840	6.026	0.2581	6.119	0.165
**39**	6.0269	5.864	0.1626	6.068	−0.0413
**40**	6.0000	6.121	−0.1213	5.939	0.0609
**42**	5.7695	5.786	−0.0166	5.831	−0.0613
**43**	5.7959	5.791	0.0051	5.737	0.059
**44**	5.7959	5.513	0.2828	5.760	0.0358
**45**	6.0315	6.176	−0.1442	6.172	−0.1406
**46**	5.6383	6.035	−0.397	5.728	−0.0898
**47**	5.8239	5.844	−0.02	5.909	−0.0852
**48**	5.5850	5.787	−0.2015	5.695	−0.1097
**49**	5.3010	5.244	0.0572	5.325	−0.0241
**50**	5.3010	5.393	−0.0917	5.275	0.026
**52**	4.7959	5.112	−0.3163	4.728	0.0682
**54**	4.7447	4.654	0.0905	4.849	−0.1045
**55**	4.7695	4.684	0.0851	4.826	−0.0568
**56**	4.8239	4.779	0.045	4.703	0.1207
**57**	4.7959	4.792	0.0037	4.902	−0.1061
**59**	4.7695	4.634	0.1357	4.657	0.1121
**60**	5.6990	5.694	0.0045	5.628	0.0712
**61**	5.6990	5.440	0.2595	5.636	0.0632
**62**	4.7959	4.760	0.0359	4.871	−0.0747
**63**	4.7695	4.812	−0.0421	4.753	0.0165
**64**	5.3010	5.582	−0.2807	5.326	−0.0249
**65**	4.8239	4.686	0.1384	4.849	−0.0248
**66**	5.7213	5.880	−0.159	5.762	−0.0407
**67**	5.6990	5.454	0.2446	5.714	−0.0154
**68**	5.6778	5.651	0.0267	5.790	−0.1123
**69**	5.8539	5.991	−0.1368	5.792	0.0624
**70**	5.7695	5.587	0.183	5.727	0.0424
**71**	5.7959	5.955	−0.1589	5.760	0.0362
**72**	6.1805	6.234	−0.0532	6.087	0.0931
**73**	6.3872	6.487	−0.1002	6.355	0.0321
**76**	5.3979	5.391	0.007	5.528	−0.1299
**77**	6.3468	6.220	0.1271	6.272	0.0749
**79**	6.6576	6.305	0.3527	6.596	0.0612
**80**	6.1135	6.136	−0.0222	6.213	−0.0992
**81**	6.3188	6.131	0.188	6.402	−0.0837
**83**	6.2291	6.107	0.1225	6.339	−0.1095
**85**	6.0000	6.025	−0.0254	6.029	−0.0285
**86**	6.3098	6.404	−0.0941	6.365	−0.0557
**89**	5.8861	6.291	−0.4053	6.112	−0.2261
**90**	6.0915	6.111	−0.0196	6.131	−0.0396
**92**	6.3566	6.171	0.186	6.281	0.0751
**93**	6.2602	6.274	−0.0138	6.251	−0.0092
**94**	6.0706	6.208	−0.1374	6.099	−0.0288
**95**	6.0410	5.983	0.0577	5.981	0.0595
**96**	6.0000	5.468	0.5325	5.981	0.0187
**97**	6.0458	6.086	−0.0406	6.038	0.0077
**98**	5.6990	5.934	−0.2348	5.638	0.0608
**99**	6.3468	6.242	0.1046	6.366	−0.0187
**100**	6.4559	6.074	0.3816	6.496	−0.0397
**105**	4.5528	4.125	0.4278	4.538	0.015
**108**	4.8239	4.736	0.0878	4.826	−0.0023
**109**	4.4949	4.403	0.0923	4.379	0.1156
**110**	4.6198	4.870	−0.2505	4.523	0.0971
**111**	4.1487	3.873	0.2756	4.082	0.0663
**112**	4.2291	4.703	−0.4742	4.182	0.0468
**113**	3.9872	4.031	−0.0437	3.984	0.0036
**117**	4.7212	4.481	0.2403	4.758	−0.0366
**119**	4.7959	4.747	0.0485	4.809	−0.0131
**121**	4.7959	4.881	−0.0849	4.889	−0.0936
**122**	4.8239	4.695	0.1286	4.871	−0.0473
**123**	4.7959	4.888	−0.0918	4.820	−0.0243
**124**	4.7695	5.019	−0.2491	4.821	−0.0511
**125**	4.7959	5.032	−0.2358	4.751	0.0454
**129**	6.3979	6.202	0.1955	6.281	0.1174
**130**	6.0458	6.086	−0.0406	6.038	0.0077
**131**	6.0000	5.468	0.5325	5.981	0.0187
**132**	6.5850	6.559	0.0255	6.608	−0.0229
**Test Set Compounds**	**pIC_50_**	**CoMFA**		**CoMSIA**	
		**Predicted**	**Residuals**	**Predicted**	**Residuals**
2 *	5.3010	5.505	−0.2035	5.296	0.0055
16 *	5.8861	5.526	0.3598	5.871	0.0147
21 *	5.4948	5.751	−0.2562	5.648	−0.1528
27 *	5.7213	5.631	0.0898	5.618	0.1034
30 *	5.585	5.522	0.0629	5.554	0.0312
35 *	5.6383	6.035	−0.397	5.728	−0.0898
41 *	5.6778	5.696	−0.0185	5.738	−0.0601
51 *	5.6990	5.581	0.1182	5.596	0.103
53 *	4.7447	4.732	0.0132	4.745	−0.0001
58 *	5.5229	5.515	0.0076	5.364	0.1585
74 *	6.1024	6.085	0.0172	6.059	0.043
75 *	5.9208	5.971	−0.0502	5.797	0.124
78 *	6.3372	6.220	0.1176	6.272	0.0654
82 *	6.4559	6.312	0.1437	6.417	0.0393
84 *	6.4948	6.451	0.0443	6.432	0.0631
87 *	6.6021	6.472	0.1298	6.523	0.079
88 *	6.0000	6.316	−0.3157	6.074	−0.0739
91 *	6.2076	6.337	−0.1291	6.360	−0.1519
101 *	3.7235	3.525	0.1989	3.684	0.0392
102 *	3.5114	3.734	−0.2221	3.580	−0.0684
103 *	3.3363	3.648	−0.3118	3.523	−0.1868
104 *	3.9586	3.937	0.0218	3.940	0.0186
106 *	4.0555	3.886	0.1694	4.104	−0.048
107 *	3.4214	3.776	−0.3549	3.506	−0.0851
114 *	4.3565	4.110	0.2468	4.286	0.0708
115 *	3.8962	3.795	0.1014	3.799	0.0972
116 *	3.9957	4.494	−0.4982	4.254	−0.2585
118 *	4.6576	4.579	0.0789	4.488	0.1701
120 *	4.9208	4.915	0.0063	4.831	0.0897
126 *	4.7959	4.816	−0.0204	4.685	0.1113
127 *	3.4089	3.904	−0.4953	3.483	−0.0741
128 *	4.8239	4.873	−0.0489	4.758	0.0663

* Test set.

**Table 3:** Chemical structures of **1**–**132** with their pIC_50_.

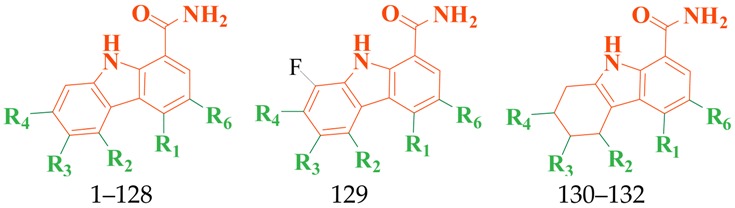							
Mol.	R_1_	R_2_	R_3_	R_4_	R_6_	IC_50_ (nM)	pIC_50_
**1**	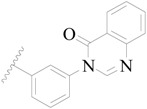	H	H	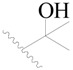	H	44	4.357
2 *	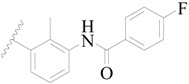	H	H	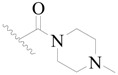	H	5.0	5.301
**3**	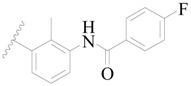	H	H	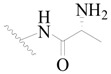	H	2.1	5.678
**4**	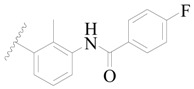	H	H	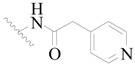	H	15	5.824
**5**	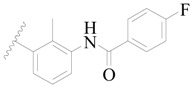	H	H	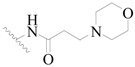	H	2.2	5.658
**6**	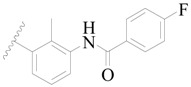	H	H	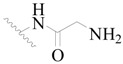	H	1.8	5.745
**7**	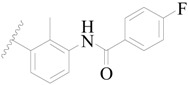	H	H	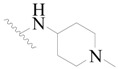	H	2.2	5.658
**8**	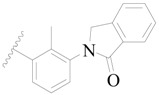	H	H	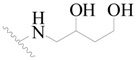	H	2.2	5.658
**9**	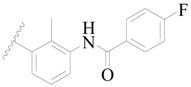	H	H	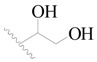	H	2.2	5.658
**10**	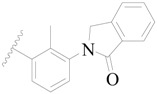	H	H	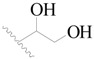	H	2.3	5.638
**11**	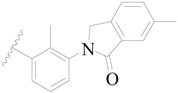	H	H	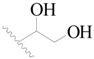	H	1.6	5.796
**12**	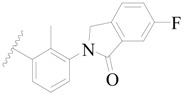	H	H	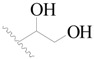	H	0.8	6.097
**13**	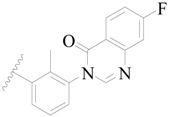	H	H	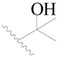	H	2.0	5.699
**14**	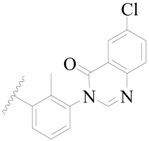	H	H	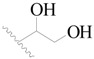	H	1.0	6.000
**15**	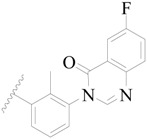	H	H	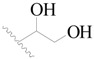	H	0.7	6.155
16 *	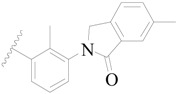	H	H	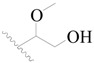	H	1.3	5.886
**17**	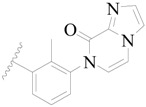	H	H	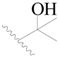	H	1.3	5.886
**18**	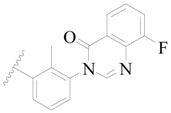	H	H	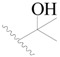	H	2.0	5.699
**19**	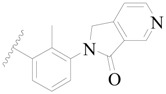	H	H	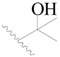	H	1.8	5.745
**20**	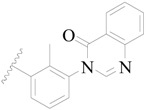	H	H	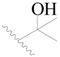	H	2.9	5.538
21 *	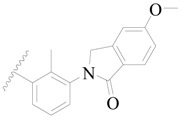	H	H	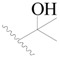	H	3.2	5.495
**22**	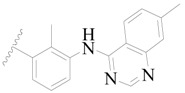	H	H	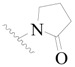	H	1.4	5.854
**23**	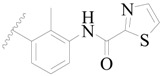	H	H	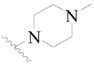	H	1.9	5.721
**24**	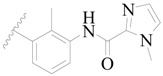	H	H	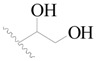	H	1.6	5.796
**25**	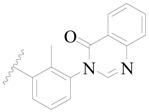	H	H	CH_2_OH	H	3.0	5.523
**26**	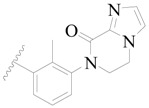	H	H	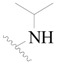	H	1.9	5.721
27 *	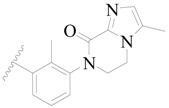	H	H	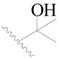	H	1.9	5.721
**28**	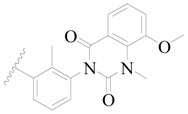	H	H	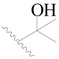	H	0.81	6.092
**29**	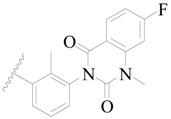	H	H	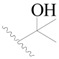	H	0.62	6.208
30 *	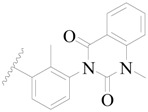	H	H	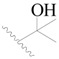	H	2.6	5.585
**31**	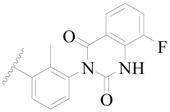	H	H	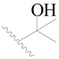	H	2.8	5.553
**32**	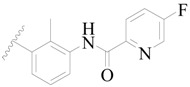	H	H	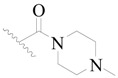	H	9.0	5.046
**33**	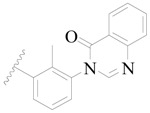	H	H	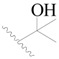	H	2.6	5.585
**34**	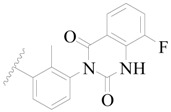	H	H	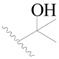	H	1.5	5.824
35 *	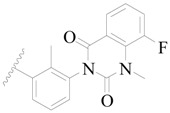	H	H	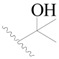	H	2.3	5.638
**36**	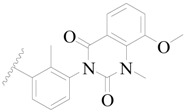	H	H	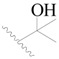	H	1.7	5.770
**37**	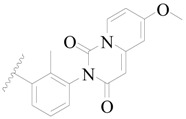	H	H	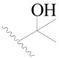	H	0.63	6.201
**38**	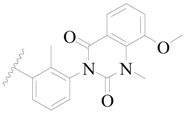	H	H	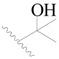	H	0.52	6.284
**39**	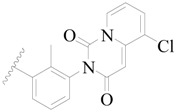	H	H	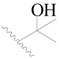	H	0.94	6.027
**40**	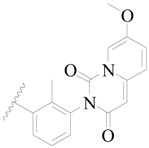	H	H	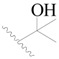	H	1.0	6.000
41 *	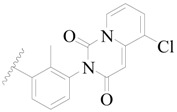	H	H	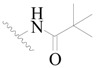	H	2.1	5.678
**42**	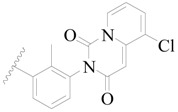	H	H	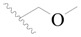	H	1.7	5.770
**43**	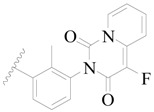	H	H	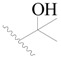	H	1.6	5.796
**44**	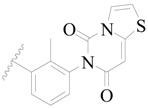	H	H	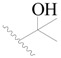	H	1.6	5.796
**45**	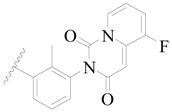	H	H	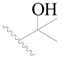	H	0.93	6.032
**46**	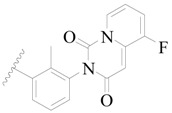	H	H	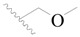	H	2.3	5.638
**47**	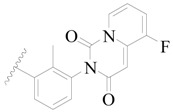	H	H	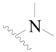	H	1.5	5.824
**48**	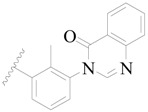	H	H	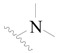	H	2.6	5.585
**49**	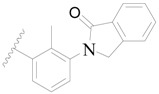	H	H	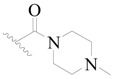	H	5.0	5.301
**50**	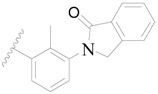	H	H	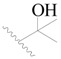	H	5.0	5.301
51 *	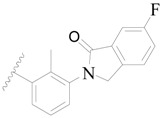	H	H	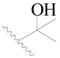	H	2.0	5.699
**52**	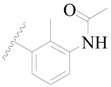	H	H	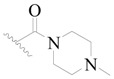	H	16	4.796
53 *	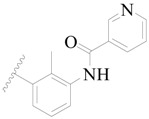	H	H	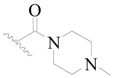	H	18	4.745
**54**	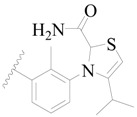	H	H	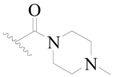	H	18	4.745
**55**	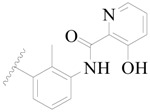	H	H	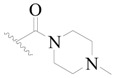	H	17	4.770
**56**	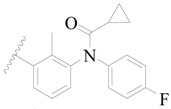	H	H	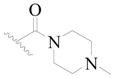	H	15	4.824
**57**	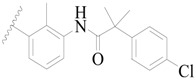	H	H	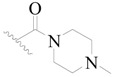	H	16	4.796
58 *	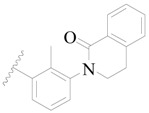	H	H	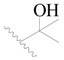	H	3.0	5.523
**59**	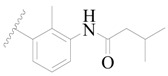	H	H	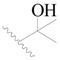	H	17	4.770
**60**	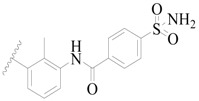	H	H	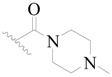	H	2.0	5.699
**61**	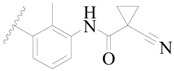	H	H	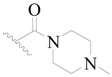	H	18	5.699
**62**	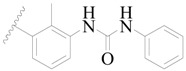	H	H	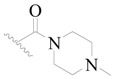	H	16	4.796
**63**	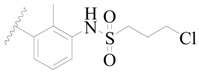	H	H	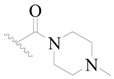	H	17	4.770
**64**	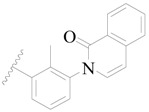	H	H	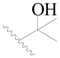	H	5.0	5.301
**65**	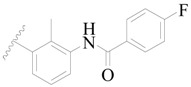	H	H	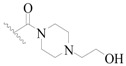	H	15	4.824
**66**	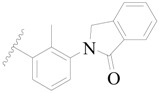	H	H	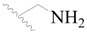	H	1.9	5.721
**67**	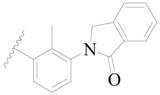	H	H	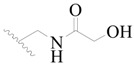	H	2.0	5.699
**68**	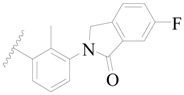	H	H	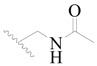	H	2.1	5.678
**69**	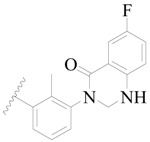	H	H	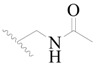	H	1.4	5.854
**70**	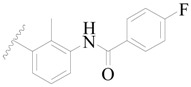	H	H	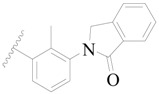	H	1.7	5.770
**71**	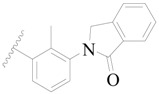	H	H	OH	H	1.6	5.796
**72**	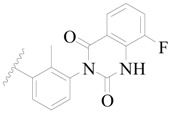	H	H	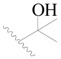	CH_3_	0.66	6.180
**73**	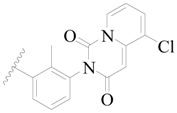	H	H	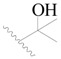	CH_3_	0.41	6.387
74 *	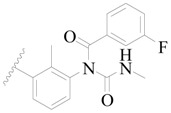	CH_3_	H	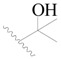	H	0.79	6.102
75 *	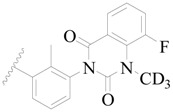	CH_3_	H	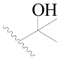	H	1.2	5.921
**76**	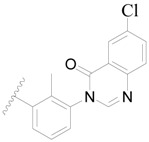	H	H	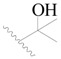	H	4.0	5.398
**77**	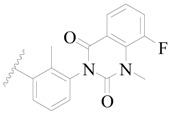	H	H	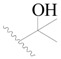	F	0.45	6.347
78 *	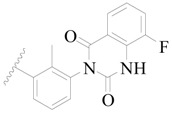	H	H	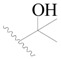	F	0.46	6.337
**79**	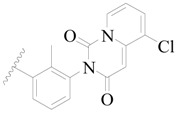	H	H	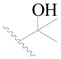	F	0.22	6.658
**80**	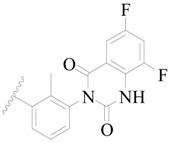	H	H	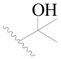	F	0.77	6.114
**81**	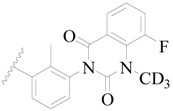	H	H	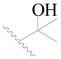	F	0.48	6.319
82 *	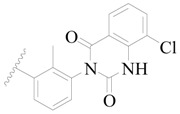	H	H	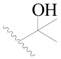	F	0.35	6.456
**83**	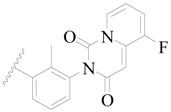	H	H	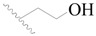	F	0.59	6.229
84 *	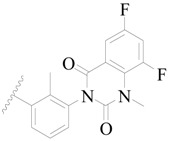	H	H	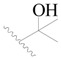	F	0.32	6.495
**85**	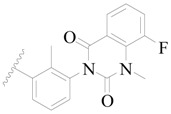	H	H	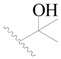	CN	1.0	6.000
**86**	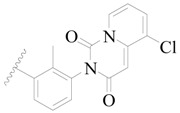	H	H	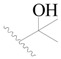	CN	0.49	6.310
87 *	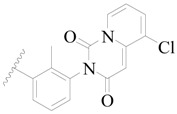	H	H	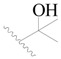	Cl	0.25	6.602
88 *	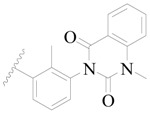	H	H	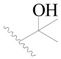	Cl	1.0	6.000
**89**	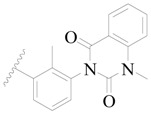	H	H	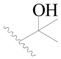	Cl	1.3	5.886
**90**	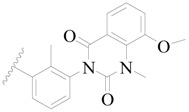	H	H	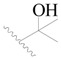	Cl	0.81	6.092
91 *	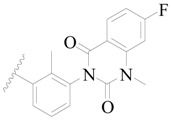	H	H	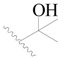	Cl	0.62	6.208
**92**	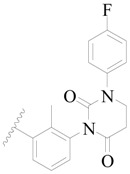	H	H	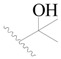	Cl	0.44	6.357
**93**	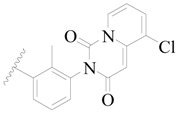	H	H	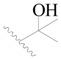	Cl	0.55	6.260
**94**	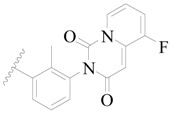	H	H	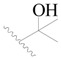	Cl	0.85	6.071
**95**	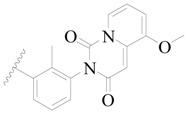	H	H	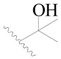	Cl	0.91	6.041
**96**	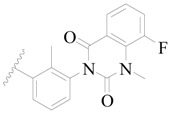	H	H	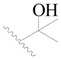	Cl	1.0	6.000
**97**	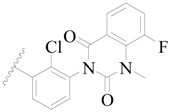	H	H	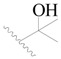	H	0.9	6.046
**98**	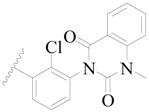	H	H	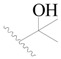	Cl	2.0	5.699
**99**	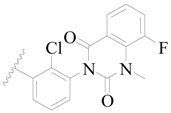	H	H	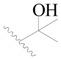	Cl	0.45	6.347
**100**	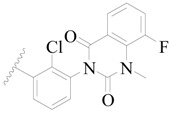	H	H	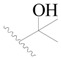	F	0.35	6.456
101 *	H	H	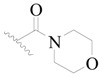	H	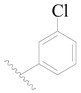	189	3.724
102 *	H	H	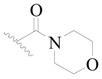	H	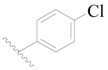	308	3.511
103 *	H	H	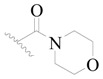	H	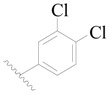	461	3.336
104 *	H	H	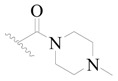	H	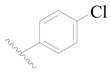	110	3.959
**105**	H	H	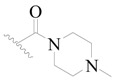	H	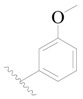	28	4.553
106 *	H	H	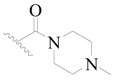	H	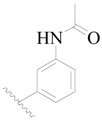	88	4.056
107 *	H	H	H	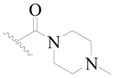	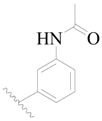	379	3.421
**108**	H	H	H	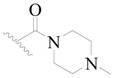	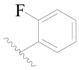	15	4.824
**109**	H	H	H	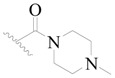	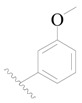	32	4.495
**110**	H	H	H	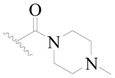	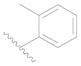	24	4.620
**111**	H	H	H	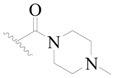	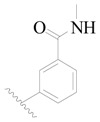	71	4.149
**112**	H	H	H	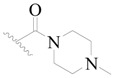	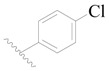	59	4.229
**113**	H	H	H	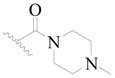	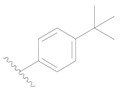	103	3.987
114 *	H	H	H	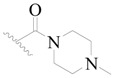	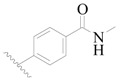	44	4.357
115 *	H	H	H	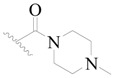	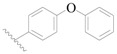	127	3.896
116 *	H	H	H	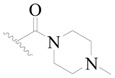	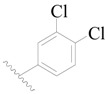	101	3.996
**117**	H	H	H	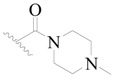	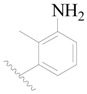	19	4.721
118 *	H	H	H	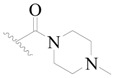	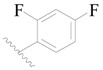	22	4.658
**119**	H	H	H	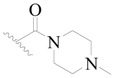	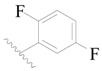	16	4.796
120 *	H	H	H	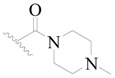	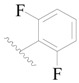	12	4.921
**121**	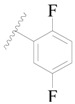	H	H	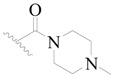	H	16	4.796
**122**	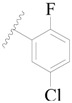	H	H	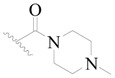	H	15	4.824
**123**	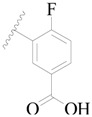	H	H	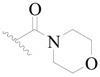	H	16	4.796
**124**	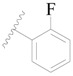	H	H	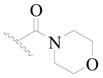	H	17	4.770
**125**	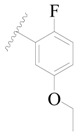	H	H	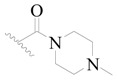	H	16	4.796
126 *	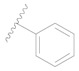	H	H	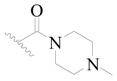	H	16	4.796
127 *	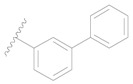	H	H	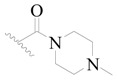	H	390	3.409
128 *	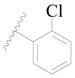	H	H	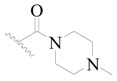	H	15	4.824
**129**	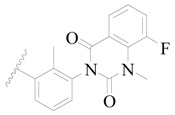	H	H	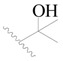	H	0.4	6.398
**130**	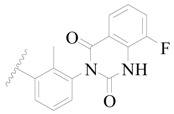	H	H	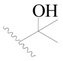	Cl	0.90	6.046
**131**	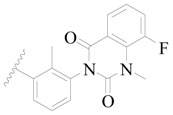	H	H	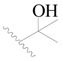	Cl	1.0	6.000
**132**	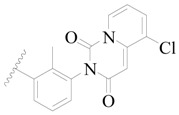	H	H	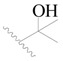	F	0.34	6.585

* Test set.

## Abbreviations

BTK	Broton’s tyrosine kinase
RA	Rheumatoid Arthritis
BCR	B-cell receptor
NSAIDs	non-steroidal anti-inflammatory drug
SAARDs	slow acting anti-rheumatic drugs
DMARDs	disease-modifying anti rheumatic drugs
3D-QSAR	three-dimensional quantitative structure–activity relationship
CoMFA	comparative molecular field analysis
CoMSIA	comparative molecular similarity indices analysis
PLS	partial least square
XLA	X-linked agammaglobulinemia
ALL	acute lymphoblastic leukemia
CML	chronic myeloid leukemia
CLL	chronic lymphocytic leukemia
